# Incidence of Carbapenem-Resistant Gram Negatives in Italian Transplant Recipients: A Nationwide Surveillance Study

**DOI:** 10.1371/journal.pone.0123706

**Published:** 2015-04-02

**Authors:** Simone Lanini, Alessandro Nanni Costa, Vincenzo Puro, Francesco Procaccio, Paolo Antonio Grossi, Francesca Vespasiano, Andrea Ricci, Sergio Vesconi, Michael G. Ison, Yehuda Carmeli, Giuseppe Ippolito, Donor-Recipient Infection (DRIn) Collaborative Study Group

**Affiliations:** 1 Department of Epidemiology and Preclinical Research, National Institute for Infectious Diseases “Lazzaro Spallanzani”, Rome, Italy; 2 Italian National Transplant Center, Italian National Institute of Health (ISS), Rome, Italy; 3 Division of Infectious Diseases, Department of Surgical and Morphological Sciences, University of Insubria-Ospedale di Circolo, Varese, Italy; 4 Direzione Generale Salute Lombardia, Regione Lombardia, Milan, Italy; 5 Divisions of Infectious Diseases & Organ Transplantation, Northwestern University Feinberg School of Medicine, Chicago, Illinois, United States of America; 6 Division of Epidemiology and the Laboratory for Molecular Epidemiology and Antibiotic Research, Sourasky Medical Center, Tel Aviv, Israel; University of California Los Angeles, UNITED STATES

## Abstract

**Background:**

Bacterial infections remain a challenge to solid organ transplantation. Due to the alarming spread of carbapenem-resistant gram negative bacteria, these organisms have been frequently recognized as cause of severe infections in solid organ transplant recipients.

**Methods and Findings:**

Between 15 May and 30 September 2012 we enrolled 887 solid organ transplant recipients in Italy with the aim to describe the epidemiology of gram negative bacteria spreading, to explore potential risk factors and to assess the effect of early isolation of gram negative bacteria on recipients’ mortality during the first 90 days after transplantation. During the study period 185 clinical isolates of gram negative bacteria were reported, for an incidence of 2.39 per 1000 recipient-days. Positive cultures for gram negative bacteria occurred early after transplantation (median time 26 days; incidence rate 4.33, 1.67 and 1.14 per 1,000 recipient-days in the first, second and third month after SOT, respectively). Forty-nine of these clinical isolates were due to carbapenem-resistant gram negative bacteria (26.5%; incidence 0.63 per 1000 recipient-days). Carbapenems resistance was particularly frequent among *Klebsiella spp*. isolates (49.1%). Recipients with longer hospital stay and those who received either heart or lung graft were at the highest risk of testing positive for any gram negative bacteria. Moreover recipients with longer hospital stay, lung recipients and those admitted to hospital for more than 48h before transplantation had the highest probability to have culture(s) positive for carbapenem-resistant gram negative bacteria. Forty-four organ recipients died (0.57 per 1000 recipient-days) during the study period. Recipients with at least one positive culture for carbapenem-resistant gram negative bacteria had a 10.23-fold higher mortality rate than those who did not.

**Conclusion:**

The isolation of gram-negative bacteria is most frequent among recipient with hospital stays >48 hours prior to transplant and in those receiving either heart or lung transplants. Carbapenem-resistant gram negative isolates are associated with significant mortality.

## Introduction

Infections remain a significant challenge to solid organ transplant (SOT).[1] In the early post transplantation period, SOT recipients are particularly vulnerable to severe bacterial infections due to the complexity of the surgical procedure,[2] use of immunosuppression,[3] prolonged hospital stay following transplantation,[4] exacerbation of pre-existing conditions[5] and, less frequently, donor transmitted pathogens.[6] Gram negative bacteria (GNs) have become a major concern recently due to the alarming spread of carbapenem resistance.[7] Carbapenem-resistant gram negative bacteria (CR-GNs) are particularly concerning because of their difficulty to treat which, in turn, results in significant morbidity and mortality, particularly among SOT recipients.[8]

CR-GNs have become particularly common in Italy[9]. A recently published report indicates that CR Enterobacteria and CR *Acinetibacter baumannii (A*. *Baumannii)* are endemic in Italy,[7] and that over 20% of *Pseudomonas aeruginosa*[10] isolates from Italian hospitals are resistant to carbapenems. To date, there have been few publications defining the epidemiology, risk factors and outcomes of CR-GNs in SOT recipients. To assess the impact of these highly resistant bacteria on SOT recipients, the Italian authority for Organ Transplants (*Centro Nazionale Trapianti; CNT*) conducted a nationwide surveillance study.

The objectives of this study were to: describe the epidemiology, assess potential risk factors and assess whether recipients with positive culture(s) to either carbapenem sucesptible GNs or CR-GNs have an increased risk of death at 90 days after SOT

## Materials and Methods

### Ethics statement

DRIn study has been approved by Ethical Committee of the Italian Health Institute (ref CE/13/382) in the framework of the national surveillance programme funded by the Italian Ministry Health. All SOT recipients signed a general consent at the time of inclusion in the waiting list. This informed consent includes acceptance of: policies for management of personal information; transplant and associated procedures; procedures for data collection and data management; policies for organs allocation and system for post-transplant surveillance. A specific consent for this study was not required as: a) information at enrollment were obtained through a mandatory national database (established by law 91/1999) which collects donors and recipients data; b)personal information was anonymized and de-identified prior to analysis; c) no intervention was planned for the purpose of the study and patients underwent medical intervention (including diagnostics) according to clinical guidelines and Italian regulations for safety and quality in solid organ transplantation.

### Setting

The CNT coordinates activities at all 44 transplant clinical units in Italy (3 of which perform SOT on pediatric patients only). In 2012 these clinical units performed 2,902 SOT (1,589 kidney, 986 liver, 231 heart, 113 lung and 68 pancreas).

### Study design

The study was designed as a cohort based on data from the national surveillance programme established by CNT to assess the burden of GNs after SOT. Patients were considered at risk from the day of transplantation until: a) 90 days thereafter or b) the day of death.

### Participant

Eligible subjects were all recipients aged ≥18 years who received SOT between 15 May and 30 September 2012 in Italy.

### Outcomes

GNs clinical isolate: a) any first GN isolate from any anatomical site; or b) a subsequent GN isolate was considered an additional event only if it was caused by a different GN species. Cultures were performed according to clinical judgment on suspicion of infection. Results of tests performed in asymptomatic subjects for surveillance purpose only (e.g. routine rectal swabs) were not included in the analysis.

CR-GNs clinical isolate: an event of GNs clinical isolates (as above) due to a GN which is phenotypically resistant to at least one carbapenem.

Death for all cause within 90 days after transplantation.

### Exposure to risk factors

Risk analysis for occurrence of clinical isolates with either GNs or CR-GNs was carried out for two groups of risk factors.

Risk factors represented by recipients’ features: sex, age (below or above the median), type of SOT (all considered as individual binary variables as a recipient may receive more than one graft type), duration of hospital stay before SOT (i.e. ≤48h or > 48h) and duration of hospital stay after SOT (as continuous variable in days).

Risk factors represented by donors’ epidemiological features: sex, age (binary below or above the median), length of stay in ICU (i.e. ≤48h or > 48h), results of cultures at the donation day (i.e.: negative for GNs, at least one carbapenems susceptible [CS] GN isolate or at least one CR-GN isolate), results of cultures during ICU stay (i.e.: negative for GNs, at least one CS GN isolate, at least one CR-GN isolate), signs of sepsis while in ICU (i.e.: no or yes); admission to an ICU where multidrug resistant (MDR) bacteria were reported in the 15 days before donation (i.e.: yes or no).

Analyses for risk of death was carried out for: recipients’ features, the results of donors’ cultures at the donation day and the occurrence of clinical isolate(s) in recipients (i.e. no infection, infection with a CS-GNs, infection with a CR-GNs).

### Data source and microbiological assessment

All data were locally collected by clinicians then sent to regional centers and eventually integrated by the CNT which also assessed data for quality and consistency.

Microbiological tests were performed in local laboratories according to national standards consistent with EUCAST criteria.[11]

According to the national guidelines[12] all donors undergo mandatory blood, urine and respiratory cultures the day of the donation. Donors’ tests during ICU stay and all recipients’ tests were performed according to clinical judgment.

### Statistical analyses

Incidence is reported as rate of events per 1000 recipient-days and incidence rate ratio (IRR) is used as measure of association between outcomes and potential risk factors. Confidence intervals are always reported with a 95% level of confidence.

Association between outcomes and potential risk factors was assessed by time adjusted and multivariate mixed-effect Poisson regression model with random intercept.[13] Time, as categorical 3-level variable (days 0–30, days 31–60 or days 61–90 after SOT) and duration of hospital stay after SOT, were considered *a priori* confounders in all multivariate analyses.

The best set of events correlation level(s) for each analysis was chosen in order to take inferential models as simple as possible and to facilitate interpretation of results, while preserving the overall robustness of results. According to these principles a specific level of event correlation was considered significant, and thus included in the analysis, only if the p-value for theta = 0 (i.e. no evidence of correlation of events) was <0.10 at least in null model (i.e. the random intercept model which include the outcome with no independent variable). Laplace approximation[14] was used for crossed-effects models. Additional file 1 describes in details how the 3 multilevel models have been set.

The best set of variables for the final multivariate model was chosen according to simplicity and fitness criteria using a stepwise approach. All variables with a IRR >1.25 or <0.80 were included in the multivariate model and the fitness of this model was compared with the full model (including all independent variables) by a likelihood ratio test (LRT). The restricted model was kept whenever LRT p-value was >0.10.

For the analysis of risk of occurrence of GNs clinical isolate(s) in recipients, interaction between all risk factors and time after SOT was assessed by LRT and the association between the outcome and risk factors was presented according to time strata whenever LRT for interaction was <0.10. Analysis of interaction between time after SOT and risk factors was not undertaken in the analyses for CR-GNs clinical isolates and mortality because of the low number of events.

Medians and interquartile ranges (IQR) were reported for time to the first onset of clinical isolate with either GNs or CR-GNs. Potential differences of median times to the first onset of clinical isolate between recipients with different baseline characteristics were assesses by Kruskal-Wallis test.

STATA 13.1 statistical package was used for all analyses. Results are reported according to the STROBE initiative.[15]

## Results

### Incidence of Gram negative isolates among Italian SOT recipients

**Fig 1:**
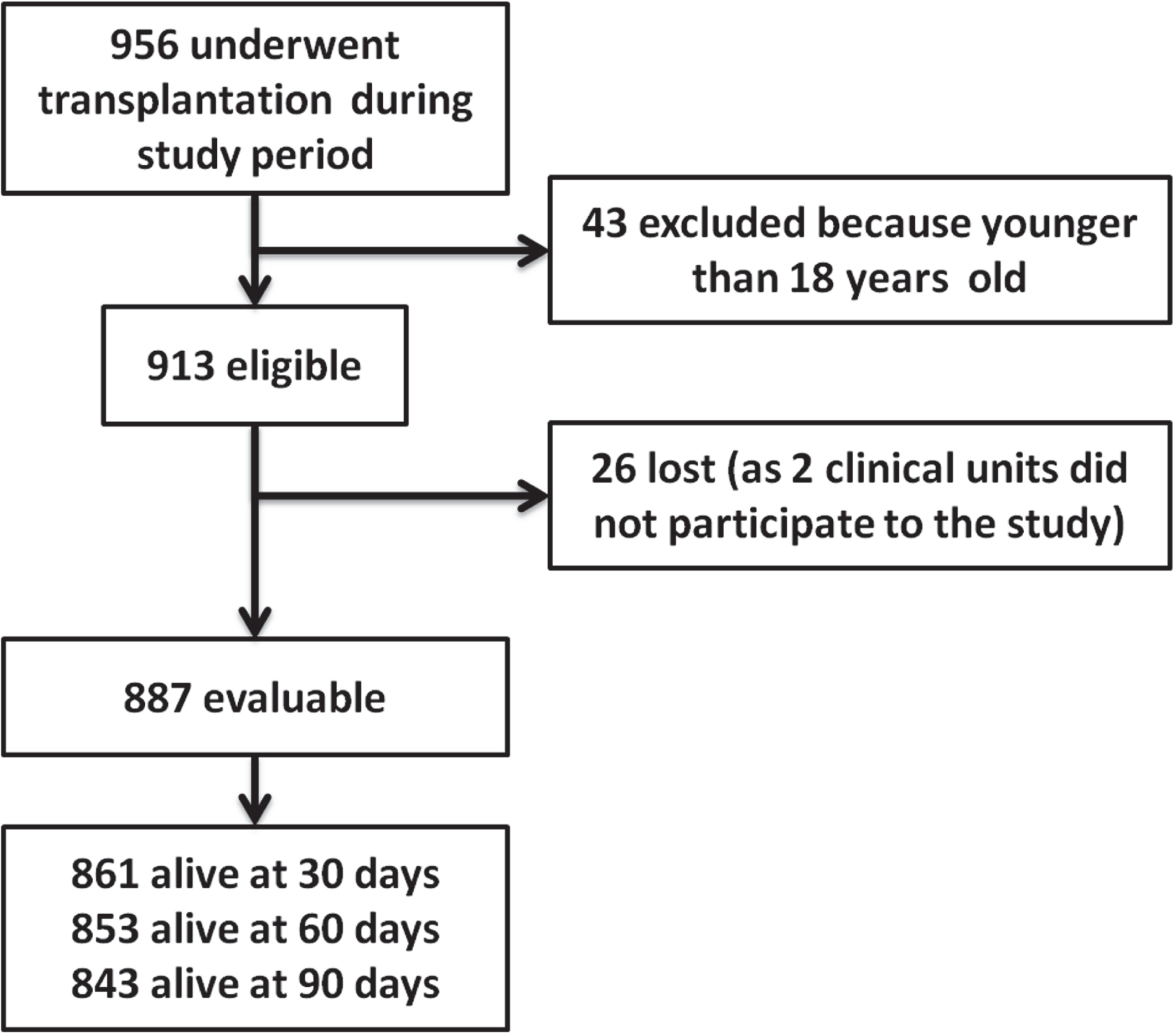
Study flowchart.

Between 15 May and 30 September 2012, 956 subjects underwent SOT in 44 clinical units. Of them 913 were eligible and 887 from 39 clinical units were analyzed (3 units performs transplantation on pediatric recipients only and 2 clinical units were excluded because provided incomplete data; see Fig 1). Among the enrolled patients, the median age was 53 and 68.9% were male.

A total of 185 GNs clinical isolates were reported in 147 recipients with a crude incidence rate of 2.39 per 1000 recipient-days (95% CI 2.07–2.77). The most common pathogens were *Escherichia coli* and *Klebsiella* spp. Forty-nine out of 185 (26.5%) isolates were resistant to carbapenems (incidence of CR-GNs 0.63 per 1000 recipient-days; 95%-CI 0.48–0.84). Remarkably the proportion of resistance to carbapenems widely varied between different species (Table 1), with *Klebsiella spp* having the highest proportion of CR-GN isolates (49.1%). On overall 44 recipients died (5.0%) by the 90^th^ day after SOT.

**Table 1:** Events of infection according the etiology (left columns) and the anatomical site (right columns).

**Bacteria**	**Carbapenem phenotype**			***Anatomical site of isolation*** ^*A*^			
	***Susceptible***	***Resistant***	***All***	***Blood***	***R*. *tract***	***U*. *tract***	***other*** ^*B*^
Klebsiella spp.	27	26 (49.1%)	53	9	7	32	12
A. baumannii	5	4 (44.4%)	9	2	5	0	2
P. aeruginosa	20	9 (31.0%)	29	3	15	7	7
E. coli	52	1 (1.9%)	53	5	7	48	6
Other enterobacteriacea	18	2(10.0%)	20	2	3	12	6
Other GN	14	7(33.3%)	21	1	8	7	3
*Total*	136	49(26.5%)	185	22	45	106	36

*Spp*. = *Specie*; *A*. *baumannii* = *Acinetobacter baumannii*; *P*. *aeruginosa* = *Pseudomonas aeruginosa*; *E*. *coli* = *Escherichia coli*; *GN* = gram negative

A) the same micro-organism may be present in different anatomical site at the time of event diagnosis, secondary involvement of multiple anatomical site was not considered

B) this includes 12 bile isolates, 7 isolates from surgical site, 8 isolate from skin infection, 3 isolate from peritoneal effusion, 5 isolate form surgical drain (kidney) 1 isolate from faeces.

### Time of onset of GNs clinical isolates

**Fig 2:**
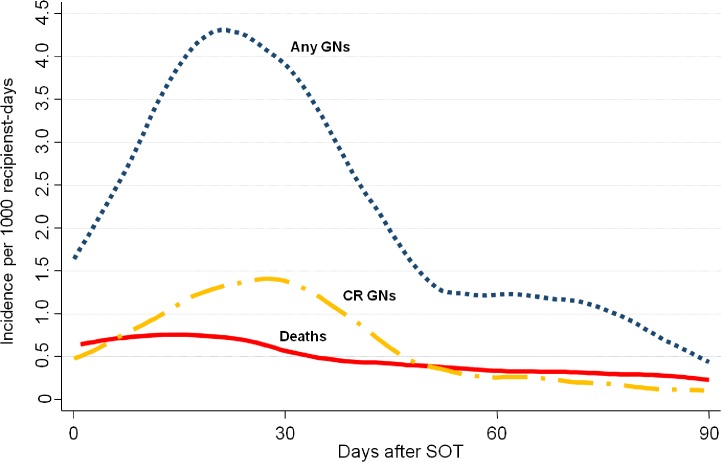
Smoothed hazard estimate for ninety days incidence rate of: infection with any gram negative bacteria (blue-dotted line; N = 185); infection with CR GN (yellow-broken line; n = 49); deaths (red-continuous line; N = 44).

The median time to the first GNs clinical isolate was 26 days (IQR 16–33; Table 2). As reported in Fig 2, GNs occurred most frequently in the early post-SOT. Incidence rates were 4.33, 1.67 and 1.14 per 1,000 recipient-days at 0–30, 31–60, and 61–90 days after SOT, respectively.

**Table 2:** Analysis for time to first infection with either any GNs or CR GNs.

**Exposure**		**Time to infection with any GNs**				**Time to infection with CR GNs**			
		***First infections***	***Median time (days)***	***IQR***	***P***	***First infections***	***Median time (days)***	***IQR***	***P***
Overall		147	26	16–33		44	24	14.5–30.5	
Recipient age	≤53	96	26	14.5–33.5		35	24	11–28	
	>53	51	26	18–32	0.653	9	24	20–34	0.493
Recipient sex	Female	47	28	20–36		17	26	11–31	
	Male	100	24	15–32	0.200	27	24	15–28	0.699
Kidney ^A^	No	74	19.5	10–30		26	20	7–28.	
	Yes	73	30	21–40	**<0.001**	18	29	21–38	**0.008**
Lung ^A^	No	136	25.5	16–32		37	24	15–30	
	Yes	11	28	25,873	0.446	7	28	11,628	0.860
Heart ^A^	No	127	28	18–35		40	24.5	16.5–31	
	Yes	20	16	5.5–21.5	**<0.001**	4	13.5	6–23	0.137
Liver ^A^	No	103	27	18–38		29	27	20–34	
	Yes	44	22.5	14–30	**0.042**	15	18	7–26	**0.047**
Pancreas ^A^	No	145	26	16–32		44	na	na	na
	Yes	2	32	18–46	0.719	0	na	na	na
Hospital stay before SOT	≤48h	137	27	17–34		38	26	18–31	
	>48h	10	17.5	5–19	**0.012**	6	6	2–18	**0.002**
Donor age	≤60	89	25	16–34		26	24.5	15–28	
	>60	58	28	18–32	0.532	18	22.5	14–36	0.738
Donor sex	female	64	27.5	15.5–34		22	27.5	11–31.	
	Male	83	24	16–33	0.645	22	23	18–28	0.323
Donor ICU stay	≤ 48 h	70	26.5	15–34		17	22	11–30	
	> 48 h	77	26	18–31	0.985	27	24	18–31	0.781
Test in ICU ^B^	no GN	134	25.5	16–34		38	24	15–31	
	CS GN	13	27	23–30	0.769	6	26	7–28	0.656
Donation day test	no GN	105	26	16–31		30	24	18–30	
	CS GN	35	25	15–36		12	19.5	8.5–31	
	CR GN	7	26	18–36	0.935	2	30.5	25–36	0.507
Donor sepsis	No	130	25.5	15–33		40	24	14.5–30.5	
	Yes	17	26	24–30	0.519	4	25.5	18–42.5	0.609
ICU with MDR	No	98	26	17–32		28	24	14.5–30.5	
	Yes	49	24	16–34	0.693	16	24	14–29.5	0.855

*IQR* = inter-quartile range; *P* = Kruskal–Wallis’s p-value; *SOT* = solid organ transplant; *ICU* = intensive care unit; *GN* = gram negative bacteria; *CS* = carbapenems susceptible; *CR* = carbapenems resistant; *MDR* = multi drug resistant organisms.

*A*) “*yes*” if recipient received the specific graft “*no*” if recipient did not (recipients may have received more than one graft at the same time)

*B*) No donor with GN-CR infection while in ICU donated organs.

GNs clinical isolates occurred significantly earlier in heart SOT (median 16 vs. 28 days p<0.001), liver SOT (median 22.5 vs. 27 days p<0.042) and in those recipients who had been admitted to hospital for more than 48h before SOT (median 17.5 vs. 27 days p<0.012). In contrast a delayed onset of GNs was observed among kidney recipients (median 30 vs. 19.5 days; p < 0.001). Donor characteristics were not associated with the time of the onset of GNs clinical isolates in recipients (Table 2).

### Time of onset of CR-GNs clinical isolates

The median time to the first CR-GNs clinical isolate was 24 days (IQR 14.5–30.5; Table 2). As reported in Fig 2, CR-GNs occurred most frequently in the early post-SOT. Incidence rates were 1.23, 0.51 and 0.16 per 1,000 recipient-days at 0–30, 31–60, and 61–90 days after SOT, respectively.

CR-GNs occurred significantly earlier in liver SOT (median 18 vs. 27 days p = 0.047) and in those recipients who had been admitted to hospital for more than 48h before SOT (median 6 vs. 26 days p = 0.002), whereas CR-GNs occurred significantly later among kidney recipients (median 29 vs. 20 days; p = 0.008). Donor characteristics were not associated with the time of CR-GN onset in recipients (Table 2).

### Risk Factors for GNs

Time adjusted models provided strong evidence that the incidence of GNs clinical isolates was significantly higher in heart and lung recipients and significantly lower in kidney recipients. Moreover we found a strong direct association between the length of hospital stay after SOT and the risk of being positive to GNs in culture. (Table 3)

**Table 3:** Analysis of the risk of infection with any GNs.

**Exposure**		**Descriptive analyses**				**Time adjusted analysis** ^A^ ^,^ ^B^		**Multivariate analysis** ^A^ ^,^ ^B^ ^,^ ^C^	
***Days*** ^B^	***Variable***	***Pop***	***event***	***RDR***	***Rate (95% CI)***	***IRR (95% CI)***	***P***	***IRR (95% CI)***	***P***
All	Overall	887	185	77,260	2.39(2.07–2.77)	-	-	-	-
All	Recipient age≤53	631	119	55,007	2.16(1.81–2.59)	base	-	-	-
	Recipient age>53	256	66	22,253	2.97(2.33–3.78)	1.24(0.87–1.75)	0.231	-	-
All	Female recipient	276	61	24,323	2.51(1.95–3.22)	base	-	-	-
	Male recipient	611	124	55,937	2.34(1.96–2.79)	0.93(0.65–1.31)	0.665	-	-
0–30	Without kidney SOT	397	68	11,473	5.93(4.67–7.52)	base	-	base	-
	With kidney SOT^**D**^	490	45	14,636	3.07(2.30–4.12)	0.46(0.30–0.71)	<0.001	0.72(0.44–1.18)	0.190
31–60	Without kidney SOT	375	14	11,158	1.25(0.74–2.12)	base	-	base	-
	With kidney SOT^**D**^	486	29	14,533	2.00(1.39–2.87)	0.83(0.39–1.77)	0.625	1.49(0.74–3.00)	0.264
61–90	Without kidney SOT	370	13	11,000	1.18(0.69–2.04)	base	-	base	-
	With kidney SOT^**D**^	483	16	14,460	1.11(0.68–1.81)	1.40(0.72–2.75)	0.322	0.97 (0.43–2.17)	0.936
All	Without lung SOT	853	171	74,632	2.29(1.97–2.66)	base	-	base	-
	With lung SOT ^**D**^	34	14	2,628	5.33(3.16–8.99)	2.56(1.31–5.01)	0.006	**2.01(1.01–4.00)**	**0.045**
0–30	Without heart SOT	816	88	24,155	3.64(2.96–4.49)	base	-	**base**	-
	With heart SOT^**D**^	71	25	1,954	12.79(8.65–18.93)	3.56(2.03–6.25)	<0.001	**2.23(1.20–4.12)**	**0.011**
31–60	Without heart SOT	797	42	23,771	1.77(1.31–2.39)	base	-	base	-
	With heart SOT^**D**^	64	1	1,920	0.52(0.07–3.70)	0.30(0.04–2.28)	0.247	0.29(0.04–2.28)	0.239
61–90	Without heart SOT	789	27	23,626	1.14(0.78–1.67)	base	-	base	-
	With heart SOT^**D**^	64	2	1,834	1.09(0.27–4.36)	0.98(0.22–4.30)	0.997	0.72(0.15–3.38)	0.673
All	Without liver SOT	591	130	51,685	2.51(2.12–2.99)	base	-	-	-
	With liver SOT ^**D**^	296	55	25,575	2.15(1.65–2.80)	0.90(0.62–1.32)	0.607	-	-
All	Without pancreas SOT	860	183	74,830	2.45(2.12–2.83)	base	-	base	-
	With pancreas SOT^**D**^	27	2	24,30	0.82(0.21–3.29)	0.36(0.08–1.58)	0.177	0.47(0.11–2.02)	0.306
All	Hosp. stay before SOT≤48h	842	171	73,771	2.32(2.00–2.69)	base	-		
	Hosp. stay before SOT >48h	45	14	3,489	4.01(2.37–6.78)	1.88(1.00–3.54)	0.051	1.39(0.74–2.02)	0.306
All	Hosp. stay after SOT	-	-	-	-	1.02(1.01–1.03)	<0.001	**1.02(1.01–1.03)**	**<0.001**
All	Donor’s age≤60	536	113	46,593	2.43(2.02–2.92)	base	-	-	-
	Donor’s age >60	351	72	30,667	2.35(1.86–2.96)	1.02(0.73–1.45)	0.890	-	-
All	Female donor	391	79	34,045	2.32(1.86–2.89)	base	-	-	-
	Male donor	496	106	43,215	2.45(2.03–2.97)	0.99(0.71–1.38)	0.964	-	-
All	Donor ICU stay≤ 48 h	489	90	43,259	2.08(1.69–2.56)	base	-	-	-
	Donor ICU stay > 48 h	398	95	34,001	2.79(2.29–3.42)	1.18(0.83–1.66)	0.356	-	-
All	Test in ICU with no GN	804	170	70,088	2.43(2.09–2.82)	base	-	-	-
	Test in ICU with CS GN ^**E**^	83	15	71,72	2.09(1.26–3.47)	1.03(0.57–1.88)	0.916	-	-
All	No GN at donation day test	649	134	56,740	2.36(1.99–2.78)	base	-	base	-
	CS GN at donation day test	205	40	17,707	2.26(1.66–3.06)	1.06(0.71–1.59)	0.777	1.16(0.78–1.73)	0.469
	CR GN at donation day test	33	11	2,813	3.91(2.17–7.06)	1.75(0.83–3.67)	0.140	2.00(0.97–4.11)	0.060
All	Donor without sepsis	792	166	69,089	2.40(2.06–2.80)	base	-	-	-
	Donor had sepsis^**F**^	95	19	8,171	2.32(1.48–3.65)	0.98(0.57–1.69)	0.950	-	-
All	ICU without MDR	663	129	57,842	2.23(1.88–2.65)	base	-	-	-
	ICU with MDR^**G**^	224	56	19,418	2.88(2.22–3.75)	1.24(0.85–1.83)	0.267	-	-
-	Days 0–30	887	113	26,109	4.33(3.60–5.20)	-	-	base	-
	Days 31–60	861	43	25,691	1.67(1.24–2.26)	-	-	**0.32(0.17–0.59)**	**<0.001**
	Days 61–90	853	29	25,460	1.14(0.79–1.64)	-	-	**0.27(0.14–0.53)**	**<0.001**

*LRT* = likelihood ratio test; *Pop* = Population; *RDR* = recipient-days at risk; *IRR* = incidence rate ratio; *P* = Wald’s test p-value; *SOT* = solid organ transplant; *ICU* = intensive care unit; *GNs* = gram negative bacteria; *CS* = carbapenems susceptible; *CR* = carbapenems resistant; *MDR* = multi drug resistant organisms; *na* = not any.

*A*) all analyses are adjusted for correlation of events at the level of clinical units, donors and recipients.

*B*) Risk analysis is provided stratified on the time for risk factors with significant interaction (i.e.: LRT for interaction ≤ 0.100).

*C*) The multivariate model was set through a stepwise forward approach to include variable with IRR>1.25 or IRR<0.80, model fitness was assessed by LRT.

*D*) recipients may have received more than one graft at the same time)

*E*) No donor with GN-CR infection while in ICU was admitted to donate organs

*F*) recipient received the graft from a donor who had sepsis any time before donation

*G*) recipient received the graft from a donor who was admitted to admission to an ICU where multidrug resistant (MDR) bacteria were reported in the 15 days before donation.

Time since SOT acted as a strong effect modifier for the risk of being positive to GNs in heart SOT (p for interaction = 0.001) and kidney SOT (p for interaction = 0.008). In fact, in both groups, the variations of IRR were statistically significant in the first month post transplantation only (Table 3). We found no evidence for statistically significant interaction between time after SOT and all other analyzed risk factors.

Multivariate model confirmed all the main findings of the time adjusted models apart from the reduced risk for GNs incidence in kidney SOT. In particular the risk of being positive for GNs increased with: duration of hospital stay after SOT, heart SOT and lung SOT (Table 3).

Estimates of IRRs and other inferential parameters (p-value and 95%CI) were simultaneously adjusted for potential correlation of events at the level of clinical units, donors and recipients (see additional file).

### Risk Factors for CR-GNs

Time adjusted models provided strong evidence that the incidence of CR-GNs clinical isolates was significantly higher among lung recipients, those recipients who had been admitted to hospital for more than 48h before SOT and recipients with longer hospital stay after SOT. Multivariate model confirmed all the main findings of the time adjusted models in addition it provided evidence for an increased risk of being positive for CR-GNs in recipients of older donors (Table 4).

**Table 4:** Analysis of the risk of infection with CR GNs.

**Exposure**		**Descriptive analyses**				**Time adjusted analysis**		Multivariate analysis ^*B*^	
		***Pop***	***event***	***RDR***	***Rate (95% CI)***	IRR(95% CI)	P	IRR(95% CI)	P
Overall		887	49	77,260	0.63(0.48–0.84)	-	-	-	-
Recipient age	≤53	631	37	55,007	0.67(0.49–0.93)	base	-	base	-
	>53	256	12	22,253	0.54(0.31–0.95)	0.69(0.33–1.43)	0.318	0.68(0.32–1.42)	0.303
Recipient sex	Female	276	18	24,323	0.74(0.47–1.17)	base	-	-	-
	Male	611	31	55,937	0.59(0.41–0.83)	0.74 ^***B***^ (0.39–1.43)	0.373	-	-
Kidney ^**C**^	No	397	28	33631	0.83(0.57–1.21)	Base	-	-	-
	Yes	490	21	43629	0.48(0.32–0.74)	0.58 ^***B***^ (0.30–1.13)	0.108	-	-
Lung ^**C**^	No	853	41	74,632	0.55(0.40–0.75)	base	-	**base**	-
	Yes	34	8	2,628	3.04(1.52–6.09)	7.17(2.61–19.65)	<0.001	**5.28(1.89–14.71)**	**0.001**
Heart ^**C**^	No	816	45	71552	0.63(0.47–0.84)	base	-	-	-
	Yes	71	4	5708	0.70(0.26–1.87)	0.99(0.31–3.15)	0.993	-	-
Liver ^**C**^	No	591	33	51,685	0.63(0.45–0.90)	base	-	-	-
	Yes	296	16	25,575	0.63(0.38–1.02)	0.89(0.45–1.77)	0.742	-	-
Pancreas ^**C**^	No	860	49	74,830	0.65(0.49–0.87)	base	-	-	-
	Yes	27	0	2,430	0.00(-)	Na	Na	-	-
Hospital stay before SOT	≤48h	842	42	73,771	0.57(0.42–0.77)	base	-	**base**	-
	>48h	45	7	3,489	2.01(0.96–4.21)	3.35(1.28–8.75)	0.013	**2.78(1.06–7.25)**	**0.037**
Hospital stay after SOT (in days)		-	-	-	-	1.03(1.02–1.04)	<0.001	**1.03(1.02–1.04)**	**<0.001**
Donor age	≤60	536	27	46,593	0.58(0.40–0.84)	base	-	**base**	-
	>60	351	22	30,667	0.72(0.47–1.09)	1.34(0.71–2.51)	0.370	**2.00(1.01–3.98)**	**0.048**
Donor sex	female	391	25	34,045	0.73(0.50–1.09)	base	-	-	-
	Male	496	24	43,215	0.56(0.37–0.83)	0.74 ^***B***^ (0.40–1.38)	0.344	-	-
Donor ICU stay	≤ 48 h	489	19	43,259	0.44(0.28–0.69)	base	-	base	-
	> 48 h	398	30	34,001	0.88(0.62–1.26)	1.78(0.93–3.39)	0.082	1.82(0.91–3.68)	0.092
Test in ICU^**D**^	no GN	804	43	70,088	0.61(0.46–0.83)	base	-	base	-
	CS GN	83	6	7,172	0.84(0.38–1.86)	1.69(0.63–4.49		1.41(0.49–4.07)	0.529
Donation day test	no GN	649	34	56,740	0.60(0.43–0.84)	base	-	base	-
	CS GN	205	13	17,707	0.73(0.43–1.26)	1.27(0.61–2.65)	0.295	1.16(0.53–2.55)	0.717
	CR GN	33	2	2,813	0.71(0.1782.84)	1.22(0.25–6.07)	0.519	1.49(0.30–7.45)	0.624
Donor has sepsis ^**E**^	No	792	45	69,089	0.65(0.47–0.87)	base	-	base	-
	Yes	95	4	8,171	0.49(0.18–1.30)	0.73(0.24–2.23)		0.68(0.21–2.17)	0.513
ICU with MDR ^**F**^	No	663	31	57,842	0.54(0.38–0.76)	base	-	base	
	Yes	224	18	19,418	0.93(0.58–1.47)	1.62(0.82–3.23)	0.167	1.36(0.68–2.74)	0.384
Time after SOT (in days)	0–30	887	32	26,109	1.23(0.87–1.73)	-	-	**base**	-
	31–60	861	13	25,691	0.51(0.29–0.87)	-	-	**0.43(0.22–0.81)**	**0.010**
	61–90	853	4	25,460	0.16(0.06–0.42)	-	-	**0.13(0.05–0.38)**	**<0.001**

*LRT* = likelihood ratio test; *Pop* = Population; *RDR* = recipient-days at risk; *IRR* = incidence rate ratio; *P* = Wald’s test p-value; *SOT* = solid organ transplant; *ICU* = intensive care unit; *GNs* = gram negative bacteria; *CS* = carbapenems susceptible; *CR* = carbapenems resistant; *MDR* = multi drug resistant organisms; *na* = not any.

*A*) all analyses are adjusted for correlation of events at the level of clinical units and recipients.

*B*)The multivariate model was set through a stepwise forward approach to include variable with IRR>1.25 or IRR<0.80, model fitness was assessed by LRT.

*C*) “*yes”* if recipient received the graft reported “*no*” if recipient did not (recipients may have received more than one graft at the same time)

*D*) No donor with GN-CR infection while in ICU was admitted to donate organs

*E*) “*yes*” if recipient received the graft from a donor who had sepsis “*no*” if recipient did not

*F*) “*yes*” if recipient received the graft from a donor who was admitted to admission to an ICU where multidrug resistant (MDR) bacteria were reported in the 15 days before donation “*no*” if recipient did not.

Estimates of IRRs and other inferential parameters (p-value and 95%CI) were simultaneously adjusted for potential correlation of events at the level of clinical units and recipients. We found no evidence for potential correlation of events at donors’ level (see additional file).

### Risk factors associated with 90-day mortality

Time adjusted and multivariate analysis provided evidence that mortality was more than 10 times higher in recipients who had culture(s) positive to CR-GNs after SOT than in those who did not. However, no evidence was found that having received organ(s) from a donor who tested positive to GNs or CR-GNs the donation day was associated to an increased risk of death (see Table 5). Higher rates of mortality were also reported for lung SOT and heart SOT, while a significant reduction of risk of death was found for kidney recipients.

**Table 5:** Analysis of mortality at 90 days

Analysis for 90-day mortality ^*A*^									
Exposure		Descriptive analyses				Time adjusted analysis		Multivariate analysis ^*B*^	
		***Pop***	***event***	***RDR***	***Rate (95% CI)***	IRR(95% CI)	P	IRR(95% CI)	P
Overall		887	44	77,260	0.57(0.42–0.77)	-	-	-	-
GN infection	No	740	25	64,855	0.39(0.26–0.57)	base	-	base	-
	CS	103	6	9,052	0.66(0.30–1.48)	1.79(0.72–4.45)	0.211	1.31(0.51–3.38)	0.575
	CR	44	13	3,353	3.88(2.25–6.68)	11.90(5.77–24.56)	<0.001	**10.23(4.69–22.31)**	**<0.001**
Recipient age	≤53	631	30	55,007	0.55 (0.38–0.78)	base	-	-	-
	>53	256	14	22,253	0.63(0.37–1.06)	1.17(0.61–2.23)	0.631	-	-
Recipient sex	Female	276	9	24,323	0.37(0.19–0.71)	base	-	base	-
	Male	611	35	55,937	0.66(0.47–0.92)	1.75(0.83–3.66)	0.138	1.80(0.83–3.92)	0.139
Kidney ^**C**^	No	397	34	33,631	1.01(0.72–1.41)	base	-	base	-
	Yes	490	10	43,629	0.23(0.12–0.43)	0.22(0.11–0.46)	<0.001	**0.43(0.19–0.99)**	**0.047**
Lung ^**C**^	No	853	38	74,632	0.51(0.37–0.70)	base	-	base	-
	Yes	34	6	2,628	2.28(1.03–5.08)	4.60(1.85–11.42)	0.001	**3.02(1.05–8.67)**	**0.040**
Heart ^**C**^	No	816	32	71552	0.45(0.32–0.63)	base	-	base	-
	Yes	71	12	5708	2.10(1.19–3.70)	5.39(2.57–11.30)	<0.001	**3.92(1.65–9.31)**	**0.002**
Liver ^**C**^	No	591	27	51,685	0.52(0.36–0.76)	base	-	-	-
	Yes	296	17	25,575	0.66(0.41–1.07)	1.21(0.64–2.31)	0.552	-	-
Pancreas ^**C**^	No	860	44	74,830	0.59(0.44–0.79)	base	-	-	-
	Yes	27	0	2,430	0.00(-)	na(na-na)	na	-	-
Hospital stay after SOT (in days)		-	-	-	-	1.01(1.00–1.03)	0.055	0.99(0.98–1.01)	0.255
Hospital stay before SOT	≤48h	842	36	73,771	0.49(0.35–0.68)	base	-	base	-
	>48h	45	8	3,489	2.29(1.15–4.58)	4.45(2.06–10.00)	<0.001	2.13(0.91–4.97)	0.081
Donation day test	no GN	649	32	56,740	0.56(0.40–0.80)	base	-	base	-
	CS GN	205	9	17,707	0.51(0.26–0.98)	0.88(0.41–1.87)	0.734	1.00(0.46–2.19)	0.995
	CR GN	33	3	2,813	1.07(0.34–3.31)	1.89(0.57–6.33)	0.301	1.37(0.39–4.81)	0.627
Time after SOT (in days)	0–30	887	26	26,109	1.00(0.67–1.46)	-	-	base	-
	31–60	861	8	25,691	0.31(0.16–0.62)	-	-	**0.36(0.16–0.80)**	**0.012**
	61–90	853	10	25,460	0.39(0.21–0.73)	-	-	**0.48(0.23–1.01)**	**0.054**

*LRT* = likelihood ratio test; *Pop* = Population; *RDR* = recipient-days at risk; *IRR* = incidence rate ratio; *P* = Wald’s test p-value; *SOT* = solid organ transplant; *ICU* = intensive care unit; *GNs* = gram negative bacteria; *CS* = carbapenems susceptible; *CR* = carbapenems resistant; *MDR* = multi drug resistant organisms; *na* = not any.

*A*) all analyses are adjusted for correlation of events at the level of clinical units.

*B*) The multivariate model was set through a stepwise forward approach to include variable with IRR>1.25 or IRR<0.80, model fitness was assessed by LRT;

*C*) “*yes”* if recipient received the graft reported “*no*” if recipient did not (recipients may have received more than one graft at the same time);

Estimates of IRRs and other inferential parameters (p-value and 95%CI) were adjusted for potential correlation of events at the level of clinical units only. We found no evidence for potential correlation of events at donors’ level (see additional file).

## Discussion

This report presents the results of the largest multicentre surveillance study specifically focused on the incidence of GNs among SOT recipients. GNs clinical isolate were common among SOT recipients in our sample (16.5% of recipients tested positive to GNs at least once with an incidence rate of 2.39 per 1000 day-person). The 26.5% of all GNs isolates were caused by CR-GN bacteria. GNs and CR-GNs occurred early after transplantation (median time to infection of 26 and 24 days, respectively) and all analyses showed a clear decreasing trend form the first to third month after SOT. This time is longer than that expected for bacterial colonization after hospitalization but, in fact, we included in the analysis only clinical isolate form cultures performed on suspicion of infection in symptomatic patients. Recognizing that GNs usually occur soon after transplantation emphasizes that efforts to identify colonization and to prevent transmission should be focused in this early time period after transplantation.

Recipients with positive culture to CR-GNs have a risk 10 times higher to die within 90 days after SOT than those who did not. The spread of CR-GN bacteria is a threat to patients’ safety globally. Others have noted the high frequencies and the associated poor outcomes of such CR-GN infections among SOT recipients.[16] This study builds on previous findings and provides a call to action for more studies to evaluate methods to limit the spread and to optimize treatment of these challenging infections among SOT recipients. In clinical units with high prevalence of CR-GNs, interventions such as enhanced recipient screening for colonization and/or enhanced isolation in both inpatient and outpatient setting could be studied with the goal of minimizing the transmission of these threatening bacteria.

The incidence of GNs and CR GNs isolates was higher in lung recipients than other SOT recipients. The increased risk of colonization and early infections with GNs in lung recipients is well known and it is potentially due to the interplay of several factors, such as: lung recipients may harbor MDR bacteria well before SOT, having received an allograft which is continuously exposed to the environment, the impaired muco-ciliary clearance, bronchial anastomotic problems, and the colonization of native lung after single-lung transplantation.[17–20]

Our study indicated that in the early post transplantation GNs were also frequently isolated among heart recipients. This observation seems to conflict with previously published evidence which indicates that early infections in heart recipients are severe but relatively infrequent events. Though speculative, we believe that this difference might be due to the effect of high antibacterial pressure to which heart recipients are exposed in the peri-transplantation period. In fact, recent published studies suggests that the risk of infection with enterobacteria increases by 4% per day of exposure to antibiotics, such as vancomycin, routinely recommended after cardiac surgery.[15,21] This hypothesis is also supported by the early onset of GN isolations we have observed. Nevertheless it cannot be excluded that this difference might have occured as we used bacterial isolations rather than infections as the primary outcome of the analysis.

The length of hospital stay either before or after SOT was independently associated with a higher risk for testing positive to CR-GN in cultures. This evidence confirms that hospital environment plays a pivotal role in the transmission of GN bacteria[4,15] and calls for interventions targeted to minimize the duration of recipients’ hospital stay before and (possibly) after SOT.

The only 2 donor dependent risk factors potentially associated with an increased incidence of GNs in recipients were having received organ from a donor who tested positive for CR-GNs the donation day (p = 0.060) and receiving organs form older donors (p = 0.048). Both observation are supported by weak evidence. In addition we could not demonstrate that any donor dependent risk factor was associated with a reduced time to first GNs clinical isolate nor did we carry out a molecular comparison between donors’ and recipients’ isolates. Therefore the study produces no conclusive evidence about the issue of donor to recipient(s) transmission.

The results of our analyses should be evaluated considering the potential limitations of the study design. The study is based on a passive national surveillance, thus local practices at the clinical unit level may have introduced bias. In fact, clinical units may have different triggers for performing cultures and/or recognizing symptoms of potential infections and as such, under-recognition and under-diagnosis could have occurred; this is less likely in more severe infections which may bias against milder or self-limited infections. The different attitude to carry out cultures may, in part, respond for the strong heterogeneity of GNs incidence between clinical units, thus we decided not to present incidence rates according to the clinical units. The study includes a short time window (138 days) therefore results might, in principle, suffer from seasonal bias or rapidly changing CR-GNs prevalence rates. The data does not include several relevant exposures, such the use of antibiotics, exposure to invasive medical devices and type of immunosuppressive protocols which have been recognized to affect the epidemiology of CR-GN bacteria. In addition to the limitations of the study itself, it must be recognized that the epidemiology of CR-GN bacteria may change rapidly because of novel introductions or effective control measures.

Despite these limitations this study represents the largest, multicenter surveillance to describe the epidemiology and risk factors for GN bacteria in SOT recipients. Even though severe, CR-GNs are, as yet, infrequent in Italian SOT recipients, in spite of the high prevalence of carbapenems resistance throughout the country. The findings of this study suggest that interventions to mitigate the impact of GNs should be directed at measures to reduce the risk of infection in the first 30 days after SOT, to optimize the peri-SOT management of lung and heart recipients and to minimize the exposure of recipients to hospital environment either before or after transplantation.

## Supporting Information

S1 File Algorithms for multilevel models definition. (DOCX)
