# Supplementary material for: Incidence of Carbapenem-Resistant Gram Negatives in Italian Transplant Recipients: A Nationwide Surveillance Study
Source: PLoS One. 2015 Apr 2;10(4):e0123706. doi: 10.1371/journal.pone.0123706 (PMC4383484; doi:10.1371/journal.pone.0123706)
Supplement: S1 File — (DOCX) [file pone.0123706.s001.docx]

### Multilevel mixed-effects Poisson regression model for infection with any GNs (Table 3)

The model contains 3 levels of nested random effects with 1 to N relation (i.e. level 0 to level 2B) and one level of crossed-effect with N to N (i.e. level 2A and level 2B). The introduction of the crossed-effect level was necessary as clinical units may receive organs from different recipients as well as organs form at same donors may be transplanted to recipients in different clinical units.

The figure below shows the algorithm used for setting the inferential model and the tests to assess significant levels of event correlation.

As reported in table 3 the final multivariate model includes:

1. 3 level of potential event correlation beside level 0; i.e. clinical units, donors and recipients);
2. 8 additional co-variates; i.e.: heart SOT (binary), lung SOT (binary), kidney SOT (binary), pancreas SOT (binary); time after SOT (categorical); results of cultural tests at the donation day (categorical); hospital stay before SOT (binary) and hospital stay after SOT (continuous);
3. 2 terms of interaction; i.e.: between time after SOT and either heart SOT or kidney SOT.

### Multilevel mixed-effects Poisson regression model for infection with CR GNs (Table 4)

This model contains 3 levels of nested random effects with N to 1 relation (i.e. level 0 to level 2B). Potential correlation of event at donors’ level were not considered according to the result of statistical tests.

The figure below shows the algorithm used for setting the inferential model and the tests to assess significant levels of event correlation (light grey for levels of event correlation which were not considered).

As reported in table 4 the final multivariate model includes:

1. 2 levels of potential event correlation beside level 0; i.e. clinical units and recipients;
2. 11 additional co-variate; i.e.: recipient age (binary); lung SOT (binary); hospital stay before SOT (binary); hospital stay after SOT (continuous); donor age (binary); donor’s length of stay in ICU (binary); donor’s cultural test results the donation day (categorical); donor’s cultural test results while in ICU (binary); having received organ for a donor who experienced sepsis (binary); having received organ for a donor who was admitted to and ICU with MDR bacteria (binary);time after SOT (categorical) .

**Multilevel mixed-effects Poisson regression model for mortality (Table 5)**

This model contains 2 levels of nested random effects with N to 1 relation (i.e. level 0 and level 2B). Potential correlation of events at recipients level was not considered for 1:1 relation between recipients and events (i.e. a death event may occur once only). Potential correlation of events at donors’ level was not considered according to the result of statistical test.

The figure below shows the algorithm used for setting the inferential model and the tests to assess significant levels of event correlation (light grey for levels of event correlation which were not considered).

As reported in table 5 the final multivariate model includes:

1. 1 level of potential event correlation beside level 0; i.e. clinical units;
2. 9 additional co-variate; i.e.: recipient’s age (binary); recipient’s sex (binary); lung SOT (binary); heart SOT (binary); kidney SOT; hospital stay before receiving SOT (binary);hospital stay after SOT (continuous); donor’s cultural test results the donation day (categorical); time after SOT (categorical).
